# Ultrasound imaging of the dorsalis pedis artery as an early indicator of the precursory changes for rheumatoid vasculitis: A case series

**DOI:** 10.1002/ajum.12373

**Published:** 2023-12-17

**Authors:** Robyn Boman, Stefania Penkala, Rosa H. M. Chan, Fredrick Joshua, Roy Tsz Hei Cheung

**Affiliations:** ^1^ School of Health Sciences Western Sydney University Campbelltown New South Wales Australia; ^2^ Translational Health Research Institute Western Sydney University Campbelltown New South Wales Australia; ^3^ Department of Electrical Engineering City University of Hong Kong Hong Kong Hong Kong; ^4^ Faculty of Medicine and Health Sciences Macquarie University Sydney New South Wales Australia

**Keywords:** adventitia, blood vessels, inflammation, intima, rheumatology

## Abstract

**Introduction:**

Clinical verification of rheumatoid vasculitis (RV) persists as a mid‐to‐late diagnosis with medical imaging or biopsy. Early and subclinical presentations of RV, in particular, can remain underdiagnosed in the absence of adequate diagnostic testing. In this study, the research demonstrated the precursory changes for RV in patients with rheumatoid arthritis (RA) using non‐invasive ultrasound imaging of a peripheral vessel.

**Method:**

Six participants were recruited: three participants with (RA) and three age‐ and gender‐matched healthy controls. All participants completed a Foot Health Survey Questionnaire (FHSQ), and participants with RA completed a Rheumatoid Arthritis Disease Activity Index‐5 (RADAI‐5). Bilateral B‐mode and Doppler ultrasound of the dorsalis pedis artery (DPA) was performed. The degree of inflammation, lumen and artery diameters, lumen diameter‐to‐artery diameter ratio and peak systolic velocity in the proximal DPA were compared between the two groups.

**Results:**

The mean RADAI‐5 score (5.4 ± 0.8 out of 10) indicated moderate disease activity amongst participants with RA. Inflammation was observed in the DPA wall in all participants with RA, compared to no inflammation observed in the control group (Friedmans two‐way analysis: χ^2^ = 15.733, P = 0.003). Differences between groups for inflammation, lumen diameter and lumen diameter‐to‐artery diameter ratio were found (P < 0.034), without differences for artery diameter and peak systolic velocity (P > 0.605). DPA wall inflammation did not correlate with FHSQ scores (*r* = −0.770, P = 0.073).

**Conclusion:**

Despite moderate RA disease activity, this is the first study to demonstrate the use of ultrasound to observe inflammation in small vessel disease. Our findings suggest ultrasound imaging may be a viable screening tool to demonstrate arterial wall inflammation, indicating the precursory changes of RV.

## Introduction

Vasculitis refers to a large group of medical conditions characterised by the pathological change in inflamed blood vessel walls.^1^ Patients who have autoimmune diseases, particularly where there is connective tissue involvement, such as rheumatoid arthritis (RA), are prone to vasculitis affecting mainly the small and medium vessels.^2^ Systemic vasculitis is a serious complication of some of these autoimmune diseases, including RA,^2^, ^3^, ^4^ with the mortality rate for one year reported to be 12% and the 5‐year mortality rate varying from 26 to 60%.^5^ Furthermore, rheumatological autoimmune diseases are increasing up to 7.1%, adding to the medical and social burden.^3^ The higher prevalence of foot problems for people with RA compared to the general population is part of this burden.^6^, ^7^ Sixty‐nine percent of people with RA, in a recent study (n = 320), reported foot and ankle pain^8^ and thus reported an unmet need for healthcare.^9^, ^10^

The burden of disease in people with RA is further increased with the coexistence of rheumatoid vasculitis (RV), including vascular symptoms of numbness, reduced foot muscle strength and skin lesions.^11^ RV management and disease progression are complicated by later diagnosis due to the current absence of early disease diagnostic screening tools.^12^

The delay in diagnosis is based on the later signs, including organ dysfunction,^13^ purpura, peripheral neuropathy, ulcers and blackened skin.^4^, ^12^ Clinical diagnosis is further complicated as typical signs such as petechiae and purpura cannot be differentiated from those seen in idiopathic, thrombocytopenia purpura, hypersensitivity vasculitis and Henoch–Schonlein purpura.^5^ The complications of RA‐associated foot pain and ulceration, with ulceration reported to be as high as 13%, attribute to the increased difficulty in RA management and concurrent RV diagnosis.^14^, ^15^

In a case report of a 61‐year‐old woman, with a 2‐year diagnosis of RA, the presentation of progressive ischaemia of the leg, associated ulcers, necrosis, infection and previous toe amputation was consistent with an advanced clinical presentation of RV and subsequently confirmed by skin biopsy.^16^ Rheumatoid vasculitis diagnosis is essential to assist differential diagnosis of more common diseases, such as peripheral vascular disease. While limb‐saving endovascular intervention is recommended for peripheral vascular disease, poorer outcomes for people with RV are reported, associated with increased vascular inflammation and coagulation reactions, resulting in limb amputation.^16^ This further highlights the need for earlier diagnosis and targeted pharmaceutical interventions.^17^

Although disease management has improved, high mortality still occurs from systemic vasculitis. Cardiac and lung complications still persist with the best‐practice use of current disease‐modifying anti‐rheumatic drugs and biological pharmaceuticals for specific management of RA without a pharmaceutical focus on RV.^5^, ^17^, ^18^ Case studies highlight the coexistence of RV early or prior to RA diagnosis.^11^, ^19^ Furthermore, the presence of symptoms that are unable to be classified is discussed as subclinical vasculitis,^20^ further complicating the diagnosis.

The increasing risk of RV presenting in the lower limb of people with RA may be linked to the reduplication of the basement membrane in the arteries to accommodate the physical needs for gravity, cooling and stasis in the legs.^4^ As histology changes are reported to occur within the arterial adventitia and intima during the vasculitic process,^4^, ^21^ the increased presence of connective tissue in the adventitia supports opportunistic inflammatory processes to advance.^21^ Duplication of the basement membrane within leg arteries provides an area of increased connective tissue for inflammatory reaction.^4^, ^21^, ^22^

Currently, tissue biopsy is the gold standard for vasculitis diagnosis but is limited in accuracy in later disease,^23^ with poor prognosis.^24^ Medical imaging is non‐invasive and can identify vasculitis in medium‐ to large‐vessel disease, but is limited to the mid to late stages of the disease.^25^, ^26^ Similarly, non‐invasive B‐mode ultrasound imaging can also assist in the diagnosis of vasculitis in medium‐to‐large arteries in the mid to late stages with the depiction of the classic halo sign.^27^ Colour and spectral Doppler changes can also demonstrate increased arterial blood velocities, which indicate further changes to the function of the artery in the mid to late stages of vasculitis.^26^, ^27^ Despite the benefits of ultrasound imaging later in the disease progression for medium‐to‐large vessels, the application to earlier disease and small vessel inflammatory changes associated with RV is lacking.

In this study, we sought to demonstrate the potential feasibility of an alternative ultrasound technique, developed by the authors, to identify and measure the early signs of inflammation in small vessel arterial walls and compare ultrasound arterial integrity parameters. The parameters used were lumen diameter, artery diameter, lumen diameter‐to‐artery diameter ratio, peak systolic velocity and inflammatory change between people with and without RA. The relationship of inflammation compared to foot pain and rheumatological pain was assessed. We hypothesised that people with RA might exhibit a greater degree of DPA wall inflammation when compared to healthy controls. We also hypothesised that symptoms measured by patient‐reported outcome measures may not be sensitive enough to associate with the early presence of DPA wall inflammation.

## Materials and methods

Six participants were recruited—three participants with RA and three age‐ and gender‐matched control participants, with all participants aged 18 years and above (Table 1). Inclusion criteria was a confirmed medical diagnosis of RA and control participants with no autoimmune diseases. Exclusion criteria included smoking, diabetes and arterial leg surgery.

**Table 1:** Characteristics of participants. Data are presented as mean and standard deviation.

	RA	Control	*P*
Age	57.0 ± 12.3	57.3 ± 11.7	0.908
Gender	3 females 0 male	3 females 0 male	1.000
Height (m)	1.64 ± 1.7	1.67 ± 2.1	0.725
Weight (kg)	69.3 ± 17.0	67.0 ± 12.5	0.429
Body mass index (kg/m^2^)	25.7 ± 6.0	23.9 ± 4.4	0.430
FHSQ	64.3 ± 9.5	92.3 ± 5.8	0.328

FHSQ, Foot Health Survey Questionnaire; RA, rheumatoid arthritis.

**Figure 1:**
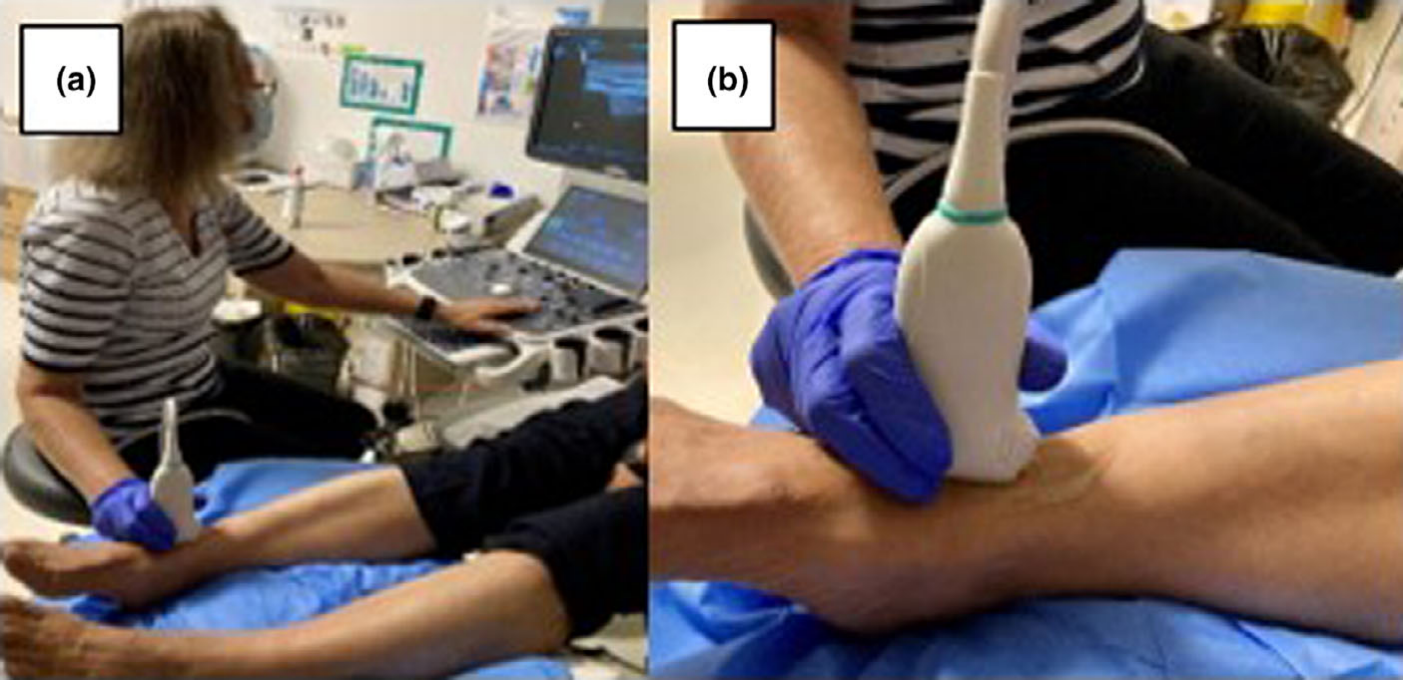
(a) Ultrasound scanning position of the proximal dorsalis pedis artery in longitudinal axis with a 13–3‐MHz linear transducer; (b) transducer positioned at proximal dorsalis pedis artery longitudinally.

One ultrasound imaging appointment was attended by each participant for the DPA assessment bilaterally. The proximal DPA was selected as the target artery for ease of access due to its superficial location at the anterior aspect of the ankle joint,^28^ and representativeness of disease in cases of small vessel systemic vasculitis. Participants were scanned by one sonographer in B‐mode (Figure 1a), using a Mindray DC‐40 ultrasound machine (Mindray Medical International Limited, Shenzhen, China) with a ≥10‐MHz linear MHz transducer (L13‐3), after resting for 10 min^29^ in a temperature‐controlled room (24.5–25°C). The image parameters were adjusted by using a different map and increasing the dynamic range to enable soft tissue changes to be observed. The proximal section of the DPA was identified with ultrasound in the longitudinal section, as the continuation of the anterior tibial artery, beginning at the distal tibial mortice (Figure 1b) and adjusted for anatomical variation.^28^, ^30^, ^31^ A slight movement of the foot was engaged to define and demonstrate the border of inflammation compared to adjacent anatomical structures.

**Figure 2:**
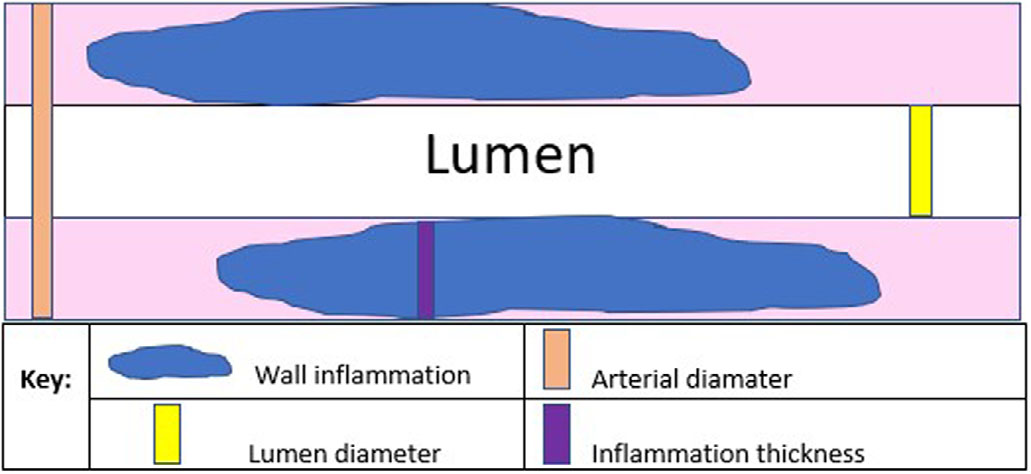
Schematic diagram of measurements within dorsalis pedis artery.

**Figure 3:**
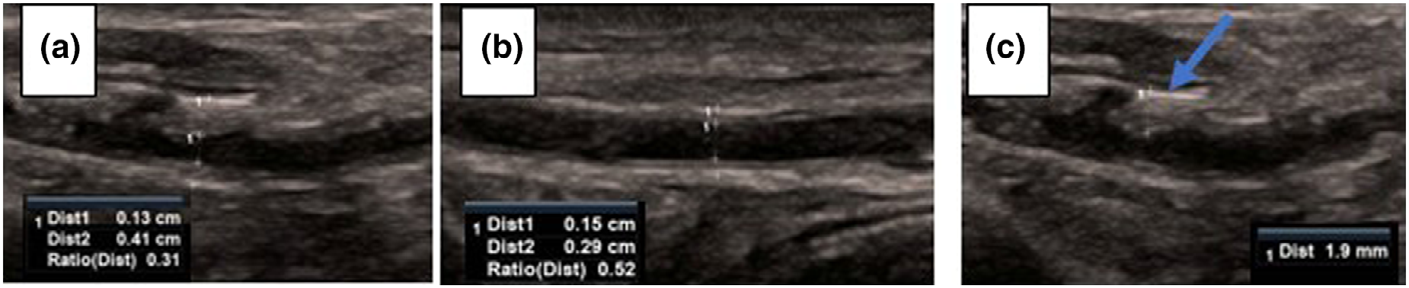
Comparison of longitudinal ultrasound image of proximal dorsalis pedis artery between a participant with rheumatoid arthritis and a control participant. (a) measured lumen and artery diameters and their ratio with areas of inflammation in participant with rheumatoid arthritis (callipers); (b) measured lumen and artery diameters and their ratio of diameters, with no evidence of inflammation in a control participant (callipers); (c) measured inflammation extending from adventitia, inflammation (callipers and arrow) in participant with rheumatoid arthritis.

**Figure 4:**
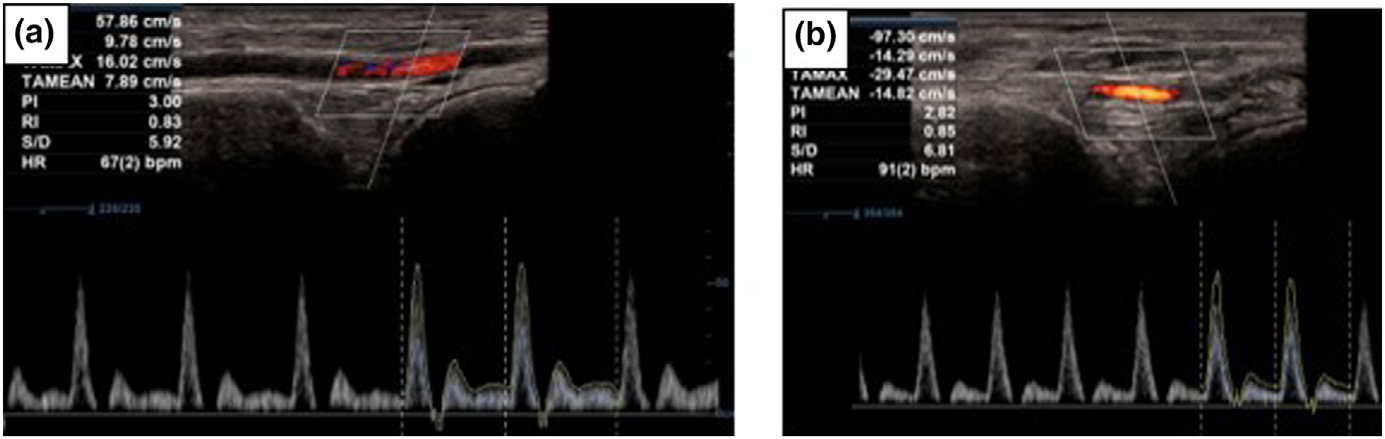
(a) Spectral trace of proximal dorsalis pedis artery for control participant; (b) Spectral trace of proximal dorsalis pedis artery for participant with rheumatoid arthritis. Normal multiwave Doppler velocity was observed for both participants.

Five parameters were scanned and measured bilaterally to assess DPA integrity from each participant and averaged for analysis. Parameters were: (i) degree of inflammation, which was defined as the thickest section of vertical height of arterial wall inflammation, including the adventitia, when inflammation was observed; (ii) lumen diameter; (iii) artery diameter; (iv) lumen diameter‐to‐artery diameter ratio; and (v) peak systolic velocity using colour and spectral Doppler (Figures 2, 3, 4).

Whilst parameters #2–4 are not routinely used for ultrasound scanning of small vessel leg arteries, we have previously demonstrated excellent intra‐ and inter‐tester reliability for static b‐mode ultrasound parameters.^32^ However, spectral Doppler (parameter #5) is a routine parameter for the assessment of blood flow,^33^, ^34^ the observation and measurement of inflammation (parameter #1) is a new technique and the focus of this study.

All participants completed demographic questions, a Foot Health Survey Questionnaire (FHSQ) and participants with RA completed the Rheumatology Arthritis Disease Activity Index‐5 (RADAI‐5) (Table 1). Both the FHSQ^35^ and RADAI‐5^36^ are reliable and validated patient‐reported outcome measures that can be used to assess foot health, quality of life and rheumatological health for people with RA.^37^ The FHSQ has four subscales, including foot pain, foot function, footwear and general foot health, with an overall score for foot health ranging from 0 (poorest) to 100 (best) (version 1.04 CareQuest).^35^ RADAI‐5 has five questions about RA health, including recent pain, joint symptoms and general health experienced ‘today’ or ‘yesterday’ and disease activity in the last 6 months.^31^ Overall scores of the RADAI‐5 range from 0 to 10; with moderate disease activity indicated by scores between 3.2 and 5.4 and scores 5.6 to 10 for high disease activity.^35^, ^38^

Due to the small sample size, data from both legs of the same participant were used, and a conservative non‐parametric Friedman test (two‐way design) was conducted to compare arterial wall inflammation, artery diameter, lumen diameter and lumen diameter‐to‐artery diameter ratio and peak systolic velocity. Significance was considered at P < 0.05. Cohen's *d* was used to quantify the effect sizes between the artery outcome measures. Effect sizes for Cohen's *d* are classified as small (0.2), medium (0.5) and large (0.8).^39^ In addition, Spearman's rank correlation tests were conducted to examine the association between artery inflammation and FHSQ.

## Ethics approval

The study has had ethical approval from the Western Sydney University ethics committee, 20 January 2022 (H14696). Written consent was obtained from each participant prior to testing. All procedures have been followed in accordance with ethical requirements.

## Results

Six female participants (n = 3 in the RA group and n = 3 in the control group) were recruited in this case series. Only female participants were recruited, as they represent the majority of people with RA.^40^ All participants with RA were on disease‐modifying anti‐rheumatic drugs and biologics, *for example* Methotrexate, Rinvoq and Cimzia. One participant was diagnosed with RA over 20 years prior; the second participant with RA was diagnosed 15–20 years ago, and the third participant was diagnosed 10–15 years ago. The mean RADAI‐5 results in our study for people with RA were 5.4 ± 0.8 out of 10, indicating moderate disease activity.^38^ Two of the three control participants were on no medication. The two groups of participants were comparable in terms of age, body height, body weight, body mass index and gender distribution (Table 1). FHSQ scores were not significantly different between the two groups (Table 1).

The RA group demonstrated inflammation in the DPA wall in comparison with the control group where no inflammation was observed (Friedmans test, χ^2^ = 15.733; df = 4; P = 0.003). The two groups demonstrated a statistical difference for inflammation (Cohen's *d* = 22.6, P = <0.001), lumen diameter (Cohen's *d* = −2.6, P = 0.034) and lumen diameter‐to‐artery diameter ratio (Cohen's *d* = −5.7, P = 0.002). There was no significant difference between the two groups for artery diameter (P = 0.815) and peak systolic velocity (P = 0.605) (Table 2). Inflammation observed when correlated with FHSQ did not demonstrate statistical significance (rho = −0.770, P = 0.073).

**Table 2:** Comparisons of ultrasound parameters between participants with and without rheumatoid arthritis. Data are presented as mean and standard deviation.

Ultrasound parameters	RA	Control	*P*	Cohen's *d*
Degree of inflammation (mm)	1.67 ± 0.2	0.00 ± 0.00	<0.001*	22.6
Lumen diameter (cm)	0.10 ± 0.02	0.17 ± 0.03	0.034*	−2.6
Artery diameter (cm)	0.32 ± 0.05	0.32 ± 0.03	0.815	−0.2
Lumen diameter/artery diameter ratio	0.31 ± 0.05	0.51 ± 0.04	0.002*	−5.7
Spectral Doppler velocity (cm/s)	74.00 ± 9.23	68.17 ± 21.25	0.605	0.5

RA, rheumatoid arthritis.

* Statistically significant difference between groups.

## Discussion

The present case series sought to demonstrate a new ultrasound imaging technique that may identify people with precursory changes for RV by evaluating the integrity of the DPA. Our findings indicated that people with RA exhibited inflammation in the DPA arterial wall. Interestingly, traditional ultrasound imaging parameters, such as artery diameter and peak systolic velocity, did not differentiate people with and without RA within this small group of participants. However, there was a statistical difference between the two groups for lumen diameter and lumen diameter‐to‐artery diameter ratio. The degree of DPA wall inflammation also did not associate with patient‐reported clinical parameters. On reflection, our ultrasound imaging technique may be a more sensitive and robust method to identify the inflammation, which may indicate the precursory changes for RV.

In accordance with our original hypothesis, we found that people with RA exhibited a greater degree of DPA wall inflammation. This ultrasound imaging technique is based on its capacity to detect thickening of the arterial wall, as depicted with the halo effect.^41^ While the halo effect has only been previously observed in medium to large vessels,^27^ our preliminary findings suggest inflammatory changes can be observed in a small artery, and the DPA is consistent with the precursory changes for RV in people with RA. Furthermore, DPA assessment could distinguish differences between those with and without RA. The inflammation identified in the DPA wall in our study is consistent with previous theory around functional adventitial pathobiology. Pathobiology discusses the duplication of the basement layer in the lower limb vessels to assist with gravity and increased pressures.^21^

Our findings are consistent with our hypothesis that precursory changes in RV can be evident prior to Doppler velocity changes. While duplex ultrasound has an important role in the assessment of peripheral vascular disease,^33^, ^34^ ultrasound has not been utilised for the early detection of precursory changes for RV. The absence of a difference between participants for peak systolic velocity is potentially due to no change in resistance of the arterial wall when inflammation is present; however, further research is required.

This new technique introduced in this study interrogates the ability to identify early arterial wall inflammatory changes within the adventitia consistent with RV, prior to clinically relevant changes seen with spectral Doppler of the arteries. The quality of life for people with RA remains difficult to assess, with the ongoing management of pain and the quality of life persisting for many people with RA.^42^ Reports of a continuous struggle to cope with the illness are documented.^43^

Through demonstrating a quantitative measurement of inflammation, a more accurate level of disease activity has the potential to improve the quality of life for people with RA and subsequent RV through improved management.

Known diagnostic evaluations for RA are frequently utilised and are established for joint evaluation. Due to the complex nature of RV diagnosis, we have incorporated a multifactorial evaluation. These evaluations include FHSQ^6^ and RADAI‐5,^44^ with the impact of RA on quality of life and, in particular, foot health well documented.^6^, ^7^, ^10^ However, these patient‐reported outcome measures have not been specifically designed for people with RV and may not be sensitive enough to detect RV symptoms, which is consistent with our findings where no significant correlation between FHSQ and arterial inflammation was found.

Moderate disease activity was reported by participants with RA at the time of the research using the RADAI‐5. This would indicate that the RADAI‐5 may not be an indicator for an earlier diagnosis of RV. While the RADAI‐5 is a validated tool for joint assessment, it is not specific to the assessment of vascular symptoms. Furthermore, it is possible that the earlier clinical symptoms of RV may be masked by RA medications that, while not specific to RV management, may add to the difficulty of RV diagnosis and earlier management.^45^ Current foot health and RA activity questionnaires may not be robust enough to detect early changes in the disease process. More specific information may need to be obtained, including non‐standard indicators such as purpura or peripheral neuropathy being present or reported.^46^ Combining these indicators with an ultrasound assessment for the presence of inflammation may assist in earlier treatment and potentially improve management for people with RA.

Although this study describes a proof‐of‐concept method to identify the precursory changes for RV by evaluating DPA parameters and wall inflammation, it is important to note a few limitations in the present study. For example, we did not have any follow‐up observations for changes or stability of inflammation. Patient‐reported outcome measures are not specific to RV. Limitations are also present with a small sample size, the observer not blinded to the diagnosis of RA and therefore at risk of bias, a single‐centre institution data source, single‐gender participants, no evidence for inflammation in the initial stages of the disease process, a restricted age range and a lack of confirmation with the gold standard biopsy. Further research with more participants may resolve these limitations.

## Conclusion

This proof‐of‐concept research indicates a potential viable and quantitative evaluation using a new ultrasound technique in people with and without arterial wall inflammation. Inflammatory changes in the arterial wall were only identified in people with RA. These inflammatory changes may be consistent with the precursory changes for RV prior to current diagnostic evaluations. This research may enhance the management of patients to monitor DPA inflammation in patients with RA and the potential concurrent diagnosis of RV. Future full‐scale research is recommended to confirm the sensitivity and specificity of this approach in the identification of people with RA with precursory changes for RV.

## Author contributions

Robyn Boman conducted the data acquisition, drafted the manuscript and created the tables and figures. Roy Tsz Hei Cheung and Stefania Penkala reviewed and edited the manuscript. Rosa H. M. Chan and Fredrick Joshua reviewed and revised the manuscript. All authors have provided final approval prior to publishing.

## Funding

No funding information is provided.

## Conflict of interest

Fredrick Joshua is an Editorial Board member of AJUM and a co‐author of this article. To minimise bias, they were excluded from all editorial decision‐making related to the acceptance of this article for publication.
